# Improvement of CO_2_-Cured Sludge Ceramsite on the Mechanical Performances and Corrosion Resistance of Cement Concrete

**DOI:** 10.3390/ma15165758

**Published:** 2022-08-20

**Authors:** Feng Xu, Rencai Chang, Dongling Zhang, Zhao Liang, Kewei Wang, Hui Wang

**Affiliations:** 1 Department of Civil Engineering, Henan Vocational College of Water Conservancy and Environment, Zhengzhou 450000, China; 2 China Railway Major Bridge Engineering Group Co., Ltd., Zhengzhou 450000, China; 3 School of Civil and Environmental Engineering, Ningbo University, Ningbo 315000, China

**Keywords:** sludge ceramsite, mechanical properties, compressive strength, water absorption rate, chloride permeability, thermal conductivity

## Abstract

The application of CO_2_ curing on sludge ceramsite may improve its mechanical properties, and then increase the corresponding corrosion resistance. In this study, the influence of CO_2_-cured sludge ceramsite on the strength and long-term properties of cement concrete is investigated. CO_2_ curing time ranges from 0 h to 2 d. The cylinder compressive strength and water absorption rate of CO_2_-cured sludge ceramsite are first determined. Additionally, the flexural and compressive strengths, the chloride permeability and the freeze—thaw damage, as well as the corresponding thermal conductivity of cement concrete, are tested. Furthermore, the corrosion resistance of reinforcement inner-sludge-ceramsite cement concrete is measured. Finally, the scanning electron microscope photos of sludge ceramsite are obtained. Results show that the cylinder compressive strength of CO_2_-cured sludge ceramsite is 15.1, ~34.2% higher than that of sludge ceramsite. Meanwhile, the water absorption rate of CO_2_-cured sludge ceramsite is 39.6, ~82.4% higher than that of sludge ceramsite. The compressive strength and the flexural strength of cement concrete with CO_2_-cured sludge ceramsite are 11.4 and 18.7, ~21.6% and ~31.5% higher than the cement concrete with sludge ceramsite, respectively. The resistance of NaCl freeze—thaw cycles, determined by comparing the mass loss rate and the loss rates of mechanical strengths, is effectively improved by CO_2_ curing, while the thermal conductivity of cement concrete is decreased by CO_2_ curing. The corrosion resistance of inner reinforcement is improved by the application of CO_2_ curing on sludge ceramsite.

## 1. Introduction

Ceramsite has been used as a lightweight aggregate in building materials for several years. Ceramsite is a material with a porous structure which shows low strength, thermal conductivity and sound transmission capacity. The addition of ceramsite can be used in the heat preservation and insulation of walls. Moreover, ceramsite usually acts as filling material for acoustic walls [1]. Furthermore, ceramsite can also be applied in road noise reduction due to its porous lightweight performance. Therefore, ceramsite is usually used in pavement concrete [2].

Previously, ceramsite was usually fired from clay. At present, sludge and excavated soil are used for sintering ceramsite [3]. However, toxic and harmful substances, such as heavy metals in sludge and excavated soil, may pollute the environment or damage human health [4]. Ceramsite concrete is light weight, and has an excellent thermal insulation effect, fire resistance and seismic performance. However, due to high water absorption and porosity, ceramsite has a poor working performance, and its strength is lower than that of ordinary concrete [5]. In order to improve the strength of traditional ceramsite and reduce the release of toxic and harmful substances from silt ceramsite, some special treatments should be carried out on the sludge or excavated soil.

Carbon neutralization and carbon peak are hot topics in today’s society [6]. Using biological methods (e.g., photosynthesis of plants), it is usually possible to reduce the content of CO_2_. However, plants cannot efficiently absorb enough CO_2_ to consume the excess CO_2_ in the atmosphere. CO_2_ curing on building materials provides ideas for its absorption [7]. Additionally, prior research pointed out that CO_2_ curing can effectively improve the mechanical strengths and the durability of cement-based materials [8,9]. Moreover, as described in some journals [10], the drying shrinkage rate of cement-based materials is decreased by CO_2_ curing.

Zhu et al. [11,12,13,14] found that CO_2_ curing can shorten the setting time of cement paste and improve mechanical strengths. Moreover, the compactness of cement’s hydration products is improved by CO_2_ curing, thus increasing the durability of cement-based materials [15]. Furthermore, as pointed out in Zhu’s research, CO_2_ curing on reinforced cement mortar can effectively improve the corresponding corrosion resistance under the external erosion of NaCl freeze–thaw cycles. Additionally, CO_2_ curing has a more obvious effect on early mechanical properties and shrinkage properties of cement-based materials. Additionally, CO_2_ curing has been used for strengthening the strength of old mortar in recycled aggregates, thus improving the mechanical strength of recycled cement concrete aggregates. Although much research about CO_2_-cured cement-based materials has been studied [16,17], little attention is paid to the research of the influence of CO_2_ curing on lightweight aggregates.

In this paper, the influence of CO_2_ curing on the cylinder compressive strength and the water absorption rate of sludge ceramsite is investigated. Additionally, the corresponding mechanical strength and anti-chloride permeability are studied. Scanning electron microscopy was carried out to research the effect of CO_2_ curing on the morphology of sludge ceramsite. This study will effectively use CO_2_ curing, and simultaneously improve the performance of lightweight aggregates. The success of this study will provide good ideas for improving silt ceramsite and reducing the content of CO_2_ in the future.

## 2. Experimental

### 2.1. Raw Materials

Sludge ceramsite was provided by Anhui Xintian Safety Environment Technology Co., Ltd., Anqing, China. The density of the sludge ceramsite was 412 kg/m^3^; moreover, the particle diameter ranged from 5 mm to 30 mm. Ceramsite sand with a density of 850 kg/m^3^ and a fineness modulus of 2.42, manufactured by Zhengzhou Yongtai ceramsite sand Co., Ltd., Zhengzhou, China, was used as a fine aggregate in this study. Ceramsite sand was dried in the blast oven until its weight was constant. The particle size of ceramsite sand ranged from 0.12 mm to 0.4 mm. The particle passing percentage of raw materials was obtained by screening experiment. The measuring process was carried out by the manufacturer. The water absorption rates of sludge ceramsite and ceramsite sand were 16% and 8%, respectively. Ordinary Portland cement, produced by Tianjin Cement Industry Company, Tianjin, China, was applied in this paper. The strength grade and the density of cement were 42.5 MPa and 3.0 g/cm^3^, respectively. Table 1 shows the main chemical composition of sludge and cement.

### 2.2. Sample Preparation

Sludge ceramsite can be produced following these steps.

Firstly, the sludge is dried and ground with loess and bentonite in proportion. Then, the raw materials are mixed into pellets and are dried at (100 ± 5) °C 6 for 2 h. When this step is finished, all mixtures are moved into the high-temperature furnace and preheated at 300 °C for 20 min. Finally, the mixtures are calcined at a high temperature of 1100 °C~1200 °C for the last 20 min.

The process for manufacturing the sludge ceramsite cement concrete can be described as follows.

Firstly, the sludge ceramsite is placed on the TH-2 digital display concrete carbonation test box produced by Shanghai Shengshi Huike testing equipment Co., Ltd., Shanghai, China for 0 h, 8 h, 16 h, 24 h, 32 h and 48 h, respectively. The CO_2_ concentration for the carbonation experiment is 8%. After carbonation, the cement, sludge ceramsite and ceramsite sand are poured into the UJZ-15 mortar mixer and mixed for 1 min. After that, water is added and mixed for another 2 min. When the mixing of materials is finished, the fresh cement concrete is poured into the mold, forming specimens with sizes of 100 × 100 × 100 mm^3^, 100 × 100 × 400 mm^3^ and Φ100 × 50 mm^3^. The JGJ/T12-2019 [18] is the Chinese standard used for manufacturing the specimens. Table 2 shows the mixing proportions of sludge ceramsite cement concrete. Ceramsite sand and ceramsite are dried in the blast oven until their weights are constant. Therefore, before preparing the specimens, the aggregates are in the dry condition.

### 2.3. Measurement Methods

#### 2.3.1. Basic Physical Properties of Sludge Ceramsite

Sludge ceramsite was firstly immersed in water for more than 2 days until a constant was maintained for the water saturated mass (mt). After that, sludge ceramsite was dried in the blast electrical oven at a temperature of 105 °C to a unified mass.

The cylindrical compressive strength of the sludge ceramsite was determined with a light-weight bearing cylinder by the following process.

The particle size of sludge ceramsite ranges from 10 mm to 20 mm, including the particle size of 10–15 mm, varying from 50% to 70%. Sludge ceramsite with these particles is used for the determination of cylindrical compressive strength. The sample is vibrated for compaction after filling in the pressure cylinder. Then, the load is conducted on the pressure cylinder with a loading rate of 0.3 kN/s~0.5 kN/s. Once the indentation depth reaches 20 mm, the pressing (*p_1_*) is recorded. These experiments are carried out according to the Chinese standard GB/T 17431.2-2010 [19].

#### 2.3.2. Mechanical Strength of Ceramsite Cement Concrete

The compressive and flexural strengths were conducted with specimen sizes of 100 × 100 × 100 mm^3^ and 100 × 100 × 400 mm^3^. The loading rates for compressive and flexural strength measurements were 0.45 MPa/s and 0.1 MPa/s, respectively. The measuring process was conducted according to the Chinese standard GBJ81-85 [20].

#### 2.3.3. The Measuring Process of Chloride Ion Permeability and Thermal Conductivity

Specimens with sizes of Φ100 × 50 mm^3^ and 100 × 100 × 100 mm^3^ were used for the determination of chloride ion permeability and thermal conductivity. The Chinese standard GB/T50082-2009 [21] was applied for the measurement of chloride ion permeability. Before the measurement of chloride ion permeability, water saturation treatment with a ZN-BSJ automatic intelligent-vacuum water-filling and fully automatic concrete vacuum water-filling tester machine, provided by Shanghai Shengshi Huike testing equipment Co., Ltd., Shanghai, China, was provided for all specimens. When the specimens were saturated with water, the chloride ion permeability was tested. Specimens with a size of 100 × 100 × 100 mm^3^ were used for measuring the thermal conductivity with a TC3000E portable thermal conductivity tester manufactured by Xi’an Xiaxi Electronic Technology Co., Ltd., Xi’an, China.

#### 2.3.4. The Corrosion Resistance of Reinforced Ceramsite Cement Concrete

**Figure 1:**
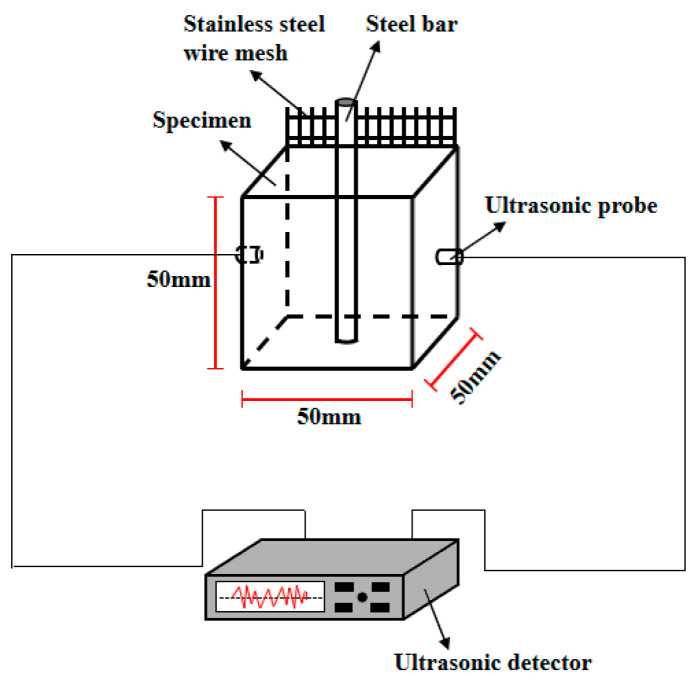
The measurement of ultrasonic velocity.

**Figure 2:**
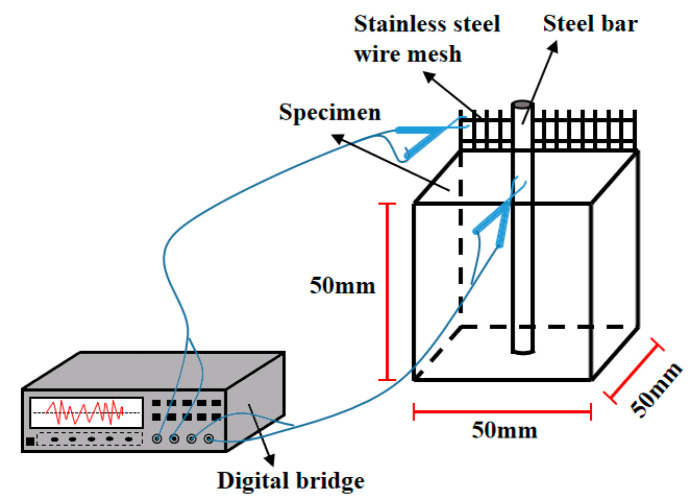
The measurement of the AC electrical resistance.

The ultrasonic velocity and AC electrical resistivity of the specimens were applied to reflect the corrosion resistance of reinforced ceramsite cement concrete. A Haichuang HC-U91 concrete ultrasonic detector provided by Xi’an Bohui Instrument Co., Ltd., Xi’an, China was used for the measurement of ultrasonic velocity. The probes were pressed on the surface of the specimens. Vaseline was smeared evenly on each size of the specimens. The AC electrical resistance was measured by the TH2810D LCR digital bridge with 104 Hz and 1V. The steel bar was imbedded in the axis position of each specimen, which served as an electrode. Meanwhile, a piece of stainless-steel mesh with an aperture of 4.75 mm and a size of 35 mm × 55 mm served as another electrode. The schematic diagrams of the ultrasonic velocity and the AC electrical resistivity are illustrated in Figure 1 and Figure 2. Equation (1) was used for the calculation of electrical resistivity, where R, A and L are the electrical resistance, the cross-section and the space between the electrodes, respectively. In this study, the space between the electrodes is 20 mm.

(1) $$\rho =\frac{RA}{L}$$

#### 2.3.5. Microscopic Characterization

The soybean size of ceramsite was taken out from the inner specimens. The selected samples were immersed in absolute ethanol for four days to prevent the hydration of cement. After that, all samples were dried in a vacuum drying oven at 60 °C for four days. The dried samples were sprayed with a gold film before measurement. After that, the SEM (Hitachi Limited., Tokyo, Japan) experiment was carried out.

## 3. Results and Discussions

### 3.1. Basic Physical Properties of Sludge Ceramsite

**Figure 3:**
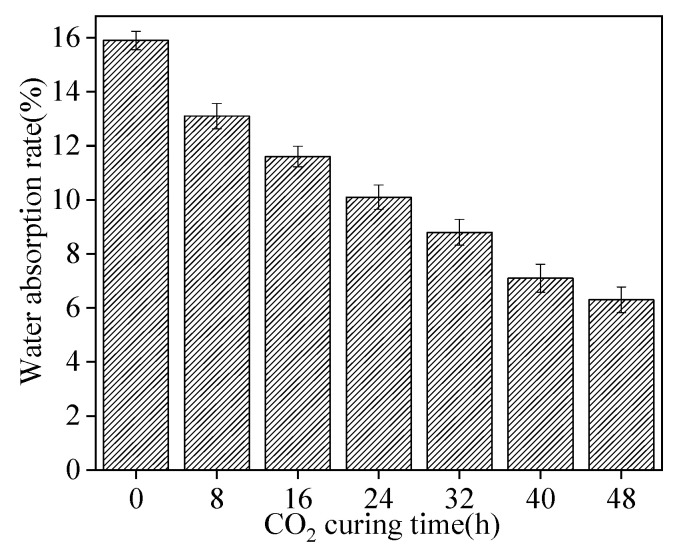
Water absorption rate of sludge ceramsite.

The water absorption rate (WAR) of sludge ceramsite, varying with the increasing CO_2_ curing time, is shown Figure 3. As illustrated in Figure 3, the WAR of sludge ceramsite decreased with the increasing CO_2_ curing time. The WAR of sludge ceramsite with CO_2_ curing for 48 h was 152.4% higher than the sludge ceramsite without CO_2_ curing. Furthermore, as depicted in Figure 3, the WAR of sludge ceramsite increased by 12.7% with the CO_2_ curing time increasing from 40 h to 48 h, which indicates that a CO_2_ curing time of 40 h was the threshold value of CO_2_ curing times. This is attributed to the fact that the oxide (Al_2_O_3_) of sludge ceramsite reacted with the CO_2_ forming the carbonate, which increased the compactness of ceramsite and decreased the WAR of sludge ceramsite [22]. Additionally, the values of the error bars were less than 0.1, showing low error and high experimental accuracy. Compared with the sludge ceramsite without CO_2_ curing, the WAR was 17.6, ~60.4% higher than the sludge ceramsite cured by CO_2_ for 8 h~48 h [23].

**Figure 4:**
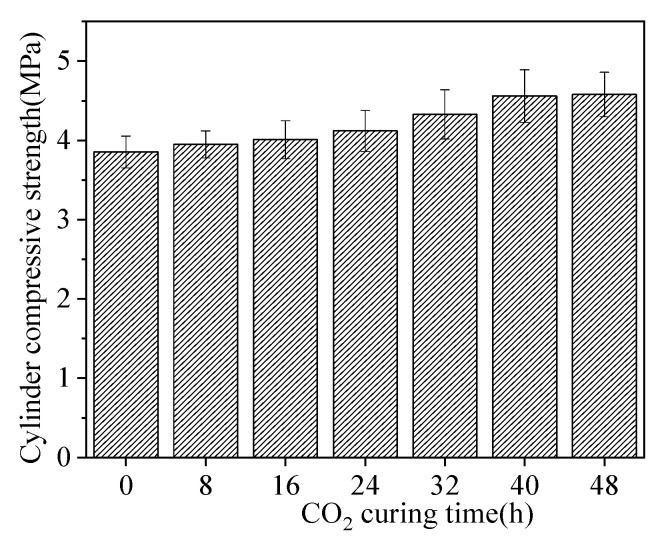
The cylinder compressive strength of sludge ceramsite.

Figure 4 illustrates the cylinder compressive strength of sludge ceramsite. As shown in Figure 4, the cylinder compressive strength of sludge ceramsite increased with the increasing CO_2_ curing time. When CO_2_ curing time increased from 0 h to 40 h, the increasing rate of cylinder compressive strength was 18.8%. However, when CO_2_ curing time increased from 40 h to 48 h, the increasing rate of cylinder compressive strength was 0.44%. This is ascribed to the fact that CO_2_ curing results in the improved compactness of sludge ceramsite [24]. Therefore, the cylinder compressive strength is improved by CO_2_ curing time. Compared with the sludge ceramsite without CO_2_ curing, the cylinder compressive strength was 2.4~18.8% higher than the sludge ceramsite cured by CO_2_ for 8 h~48 h [23].

### 3.2. The Slump of Sludge Ceramsite Cement Concrete

**Figure 5:**
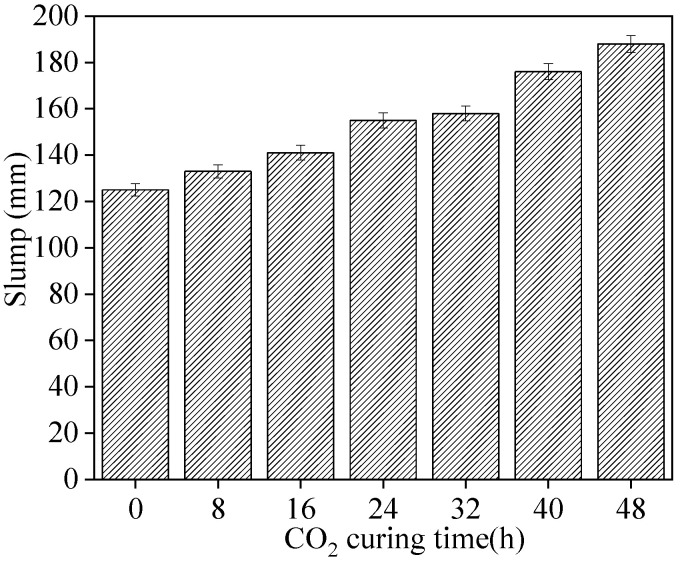
The slump of fresh sludge ceramsite cement concrete.

Figure 5 presents the slump of fresh sludge ceramsite cement concrete. The aggregates (sludge ceramsite) were in a saturated-surface dry condition during preparation of the concrete. As observed in Figure 5, the slump of fresh sludge ceramsite cement concrete increased with CO_2_ curing time. This is ascribed to the fact that the application of CO_2_ curing on sludge ceramsite is effective in decreasing its pores [25]. Therefore, the water absorption capacity was decreased by the CO_2_ curing time, eventually leading to an increase in the slump of fresh sludge ceramsite cement concrete.

### 3.3. The Mechanical Strengths of Sludge Ceramsite Cement Concrete

**Figure 6:**
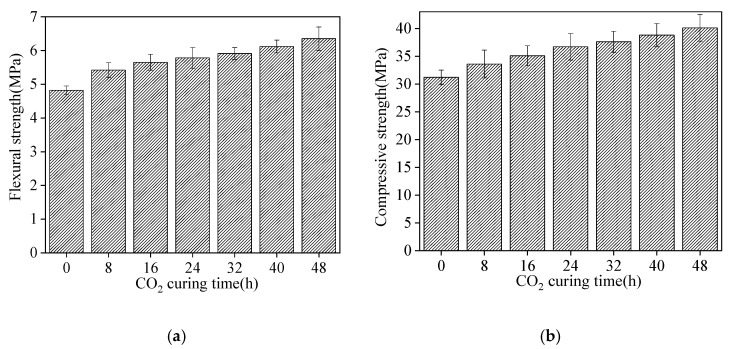
The mechanical strengths of sludge ceramsite cement concrete. (**a**) The compressive strength. (**b**)The flexural strength.

The mechanical strengths of sludge ceramsite cement concrete are illustrated in Figure 6. As shown in Figure 6, the flexural and compressive strengths of sludge ceramsite cement concrete increased with the increasing CO_2_ curing time. When CO_2_ curing time ranged from 0 h to 48 h, the increasing rate of flexural strength increased from 0% to 31.7%. Meanwhile, the increasing rate of compressive strength varied from 0% to 28.5%. This is attributed to the fact that CO_2_ curing can improve the compactness of sludge ceramsite, thus increasing the flexural and compressive strengths of sludge ceramsite cement concrete [26]. Additionally, the values of error bars were lower than 0.15, indicating low values of error bars.

### 3.4. The Damage of NaCl Freeze–Thaw Cycles

**Figure 7:**
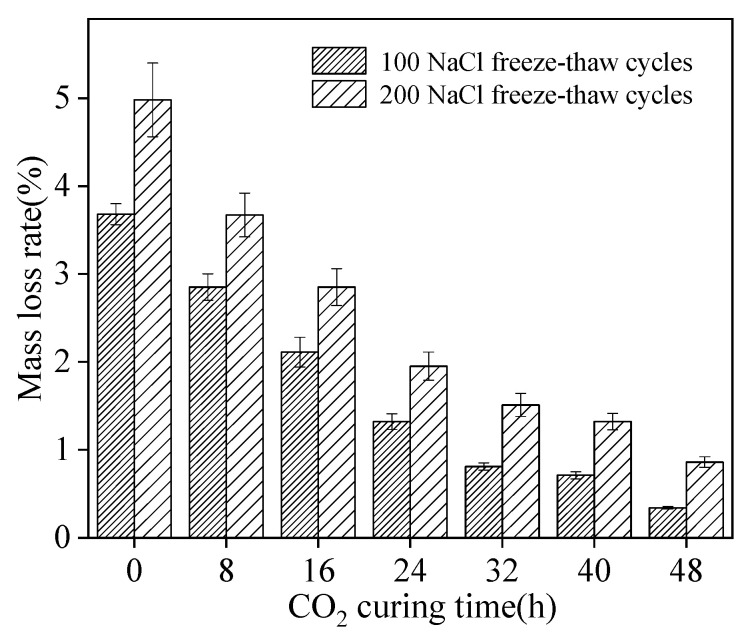
The mass loss rate of sludge ceramsite cement concrete.

The mass loss rate of sludge ceramsite cement concrete is shown in Figure 7. As depicted in Figure 7, the mass loss rate was decreased by the increasing curing time and was increased by higher NaCl freeze–thaw cycles. This is ascribed to the fact that the CO_2_ curing effectively improves the compactness of sludge ceramsite cement concrete [27,28,29]. Therefore, when the NaCl freeze–thaw cycles were acted upon the specimens, the less mass on the surface of specimens spalled. The values of the error bars were lower than 0.2, indicating high accuracy of experimental results.

**Figure 8:**
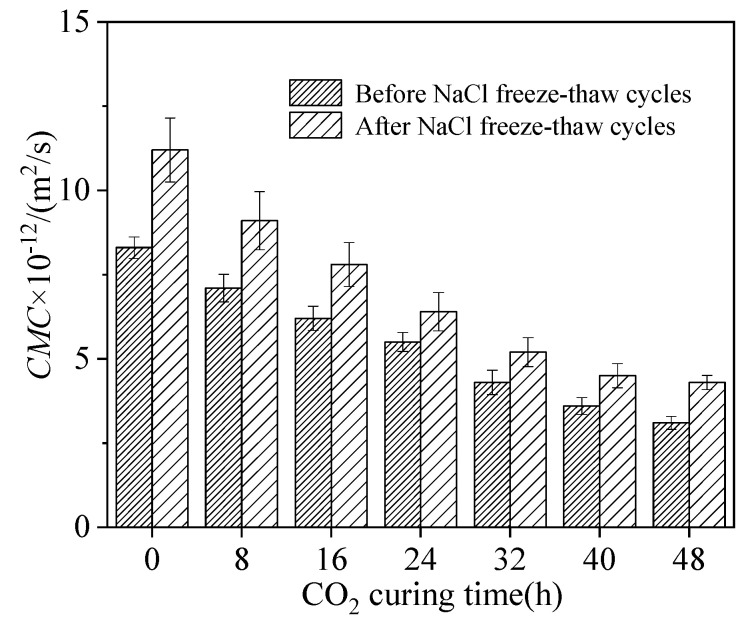
The chloride ion permeability coefficient of sludge ceramsite cement concrete.

Figure 8 illustrates the chloride ion permeability coefficient of sludge ceramsite cement concrete. The experiments were carried out after the specimens were standard cured for 28 days, or after the specimens experienced 300 NaCl freeze–thaw cycles. As depicted in Figure 8, the chloride ion permeability coefficient of sludge ceramsite cement concrete increased with the increasing number of NaCl freeze–thaw cycles and decreased with CO_2_ curing time. This is attributed to the fact that the increasing number of NaCl freeze–thaw cycles increase the expansion of internal cracks in concrete, which increases the chloride ion permeability coefficient [30]. Meanwhile, CO_2_ curing increases the compactness of the material and delays the expansion of cracks. Therefore, the chloride ion permeability coefficient of sludge ceramsite cement concrete decreased with the increasing CO_2_ curing time [31,32].

**Figure 9:**
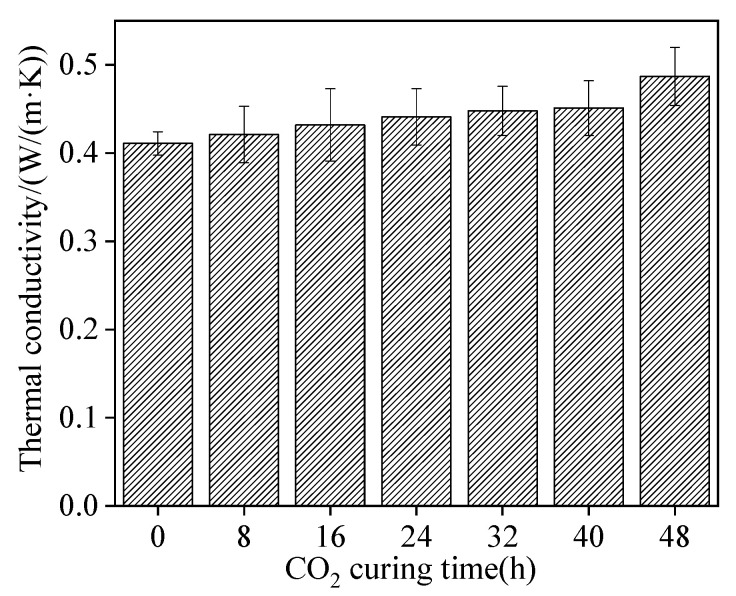
The thermal conductivity of sludge ceramsite cement concrete.

Figure 9 shows the thermal conductivity of sludge ceramsite cement concrete. It can be found in Figure 9 that the thermal conductivity increased with increasing CO_2_ curing time. This is due to the fact that CO_2_ curing on the sludge ceramsite can improve the compactness [33]. Meanwhile, higher compactness of solid leads to high thermal conductivity [34]. Additionally, 48 h CO_2_ curing on the sludge ceramsite increased the thermal conductivity by 18.5%. Therefore, the thermal conductivity varied inapparently with CO_2_ curing.

### 3.5. The Corrosion Resistance of Inner Steel Bars

When steel bars corrode, the rust on the surface results in the cracks of inner specimens. The ultrasonic velocity of reinforced sludge ceramsite cement concrete can be used to reflect the following corrosion. Moreover, when steel bars corrode, the rust on the steel bars’ surface prevents the migration of electrons, leading to an increase in electrical resistance. Therefore, the electrical resistance can be applied in the corrosion resistance of steel bars. The ultrasonic velocity and electrical resistivity of specimens cured in a standard environment for 28 days, or after 300 NaCl freeze–thaw cycles, were applied to reflect the corrosion resistance of reinforced sludge ceramsite cement concrete. The NaCl freeze–thaw cycles were carried out following the steps. After standard curing for 24 days, some specimens are immersed in the solution containing 3% NaCl for 4 days, and are moved to the rapid freezing and thawing test box with working temperatures of −15 °C~8 °C.

**Figure 10:**
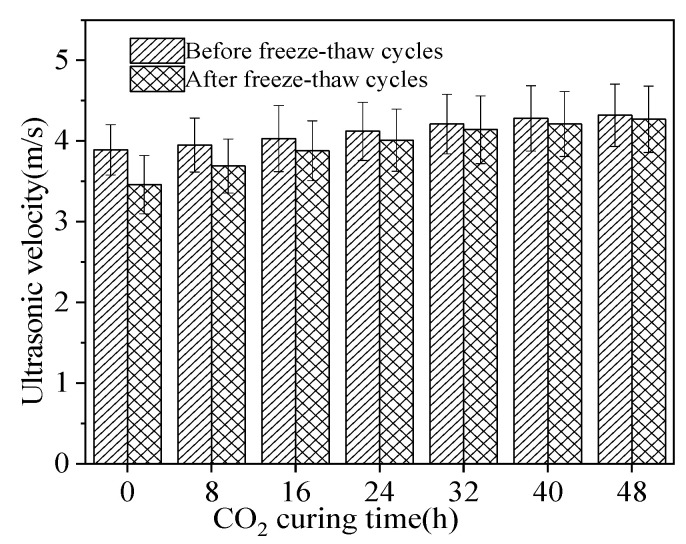
The ultrasonic velocity of reinforced sludge ceramsite cement concrete.

Figure 10 shows the ultrasonic velocity of reinforced sludge ceramsite cement concrete. It can be observed from Figure 10 that the ultrasonic velocity of reinforced sludge ceramsite cement concrete increased with increasing CO_2_ curing time. This is attributed to the fact that CO_2_ curing can make ceramsite denser, thus increasing resistance to chloride penetration [35]. Hence, the corrosion resistance of reinforced sludge ceramsite cement concrete is improved by the application of CO_2_ curing on sludge ceramsite. Meanwhile, the NaCl freeze–thaw cycles led to a decrease in the ultrasonic velocity. This is ascribed to the fact that frost heave stress and chloride ion erosion caused by NaCl freeze–thaw cycles leads to increasing cracks in inner specimens [36]. Consequently, the ultrasonic velocity of reinforced sludge ceramsite cement concrete was decreased by NaCl freeze–thaw cycles.

**Figure 11:**
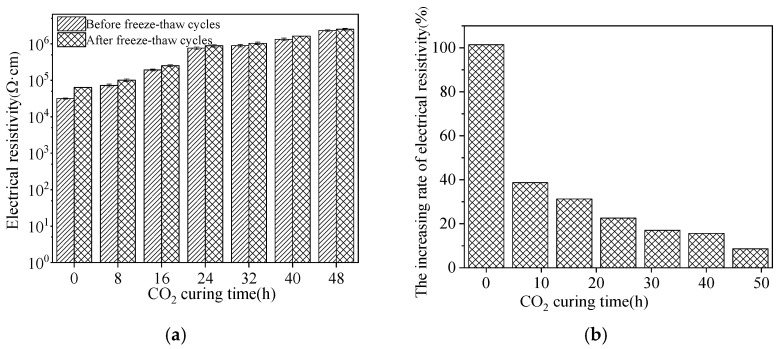
The electrical resistivity of reinforced sludge ceramsite cement concrete. (**a**) The electrical resistivity. (**b**) The increasing rate of electrical resistivity.

The electrical resistivity and the following increasing rate of reinforced sludge ceramsite cement concrete is illustrated in Figure 11. It can be observed in Figure 11 that the electrical resistivity of reinforced sludge ceramsite cement concrete increased with increasing CO_2_ curing time. The increasing rate of electrical resistivity induced by NaCl freeze–thaw cycles was decreased by the application of CO_2_ curing on sludge ceramsite. This research findings proves that the application of CO_2_ curing on sludge ceramsite can improve the corrosion resistance of reinforcement [37,38,39,40,41,42].

**Figure 12:**
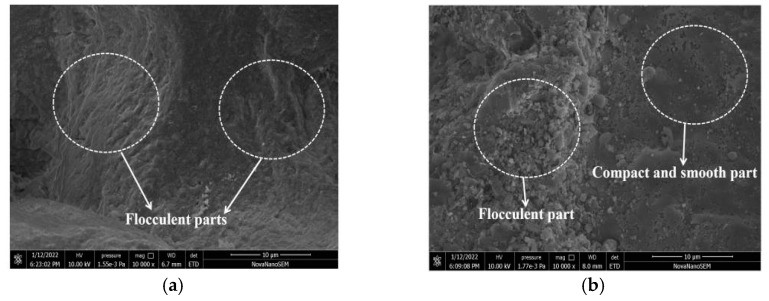
SEM microstructure photos of specimens. (**a**) Without CO_2_ curing. (**b**) CO_2_ curing for 48 h.

The SEM microstructure photos of ceramsite cured in CO_2_ for 0 h and 48 h, respectively, are illustrated in Figure 12. As depicted in Figure 12, ceramsite without CO_2_ curing possessed more flocculent parts. Meanwhile, when ceramsite was cured in CO_2_ for 48 h, more dense parts were found in ceramsite.

## 4. Conclusions

This paper provides a method (CO_2_ curing on sludge ceramsite) to improve the mechanical properties of silt ceramsite. After investigating the influence of CO_2_-cured sludge ceramsite on the properties of cement concrete, the conclusions can be summarized as follows.

The application of CO_2_ curing on sludge ceramsite can increase the cylinder compressive strength by 15.1~34.2% and decrease the water absorption rate by 39.6~82.4%. This reflects the fact that CO_2_ curing on sludge ceramsite can improve its compactness.

The addition of CO_2_-cured sludge ceramsite can effectively increase the mechanical strength of cement concrete. Cement concrete with CO_2_-cured sludge ceramsite showed higher compressive strength and the flexural strength than that with normal sludge ceramsite. The increasing rates of the compressive strength and the flexural strength were 11.4~21.6% and 18.7~31.5%.

The NaCl freeze–thaw cycles’ resistance to sludge ceramsite cement concrete was improved by CO_2_-cured sludge ceramsite. The thermal conductivity of ceramsite cement concrete was decreased by the addition of CO_2_-cured sludge ceramsite. The corrosion resistance of inner steel bars was increased by the application of CO_2_ curing on sludge ceramsite.

## Figures and Tables

**Table 1:** Chemical composition of the cementitious material (%).

Types	SiO_2_	Al_2_O_3_	Fe_x_O_y_	MgO	CaO	SO_3_	ZnO	R_2_O	Loss on Ignition
Sludge	69.75	15.18	5.91	2.31	2.43	-	-	2.14	2.28
P·O cement	20.9	5.5	3.9	1.7	62.2	2.7	-	-	3.1

**Table 2:** Mix proportions of ceramsite per one cubic meter (kg).

Cement	Water	Ceramsite Sand	Ceramsite	Water-Reducing Agent	CO_2_ Curing Time (h)
500	200	700	300	0.5	0
500	200	700	300	0.5	8
500	200	700	300	0.5	16
500	200	700	300	0.5	24
500	200	700	300	0.5	32
500	200	700	300	0.5	48

## Data Availability

The data used to support the findings of this study are available from the corresponding author upon request.

## References

[1] Zhai X., Yan J., Cao C. (2021). Seismic performance and flexible connection optimization of prefabricated integrated short-leg shear wall filled with ceramsite concrete. Constr. Build. Mater..

[2] Shen Y., Huang J., Ma X., Hao F., Lv J. (2020). Experimental study on the free shrinkage of lightweight polymer concrete incorporating waste rubber powder and ceramsite. Compos. Struct..

[3] Cheng G., Li Q., Su Z., Sheng S., Fu J. (2018). Preparation, optimization, and application of sustainable ceramsite substrate from coal flfly ash/waterworks sludge/oyster shell for phosphorus immobilization in constructed wetlands. J. Clean. Prod..

[4] Monteiro S., Alexandre J., Margem J., Sánchez R., Vieira C. (2008). Incorporation of Sludge Waste from Water Treatment Plant into Red Ceramic. Constr. Build. Mater..

[5] Yang Z., Ji R., Liu L., Wang X., Zhang Z. (2018). Recycling of municipal solid waste incineration by-product for cement composites preparation. Constr. Build. Mater..

[6] Kongboon R., Gheewala S.H., Sampattagulad S. (2022). Greenhouse gas emissions inventory data acquisition and analytics for low carbon cities. J. Clean. Prod..

[7] Xian X., Zhang D., Lin H., Shao Y. (2022). Ambient pressure carbonation curing of reinforced concrete for CO_2_ utilization and corrosion resistance. J. CO_2_ Util..

[8] Kamal N.L.M., Itam Z., Sivaganese Y., Beddu S. (2020). Carbon dioxide sequestration in concrete and its effects on concrete compressive strength. Mater. Today Proc..

[9] Cao H., Liang Z., Peng X., Cai X., Wang K., Wang H., Lyu Z. (2022). Research of Carbon Dioxide Curing on the Properties of Reactive Powder Concrete with Assembly Unit of Sulphoaluminate Cement and Ordinary Portland Cement. Coatings.

[10] Sirtoli D., Wyrzykowski M., Riva P., Tortelli S., Marchi M., Lura P. (2019). Shrinkage and creep of high-performance concrete based on calcium sulfoaluminate cement. Cem. Concr. Compos..

[11] Zhu J., Qu Z., Liang S., Li B., Du T., Wang H. (2022). The macro-scopic and microscopic properties of cement paste with carbon dioxide curing. Materials.

[12] Witoon T., Lapkeatseree V., Numpilai T., Cheng C., Limtrakul J. (2022). CO_2_ hydrogenation to light olefifins over mixed Fe-Co-K-Al oxides catalysts prepared via precipitation and reduction methods. Chem. Eng. J..

[13] Witoon T., Numpilai T., Nijpanich S., Chanlek N., Kidkhunthod P., Cheng C.K., Ng K.H., Vo D.-V.N., Ittisanronnachai S., Wattanakit C. (2022). Enhanced CO_2_ hydrogenation to higher alcohols over K-Co promoted In_2_O_3_ catalysts. Chem. Eng. J..

[14] AL-Ameeri A.S., ImranRafifiq M., Tsioulou O., Rybdylova O. (2021). Impact of climate change on the carbonation in concrete due to carbon dioxide ingress: Experimental investigation and modelling. J. Build. Eng..

[15] Atsbha T.G., Zhutovsky S. (2022). The effect of external curing methods on the development of mechanical and durability-related properties of normal-strength concrete. Constr. Build. Mater..

[16] Qin L., Gao X., Chen T. (2019). Influence of mineral admixtures on carbonation curing of cement paste. Constr. Build. Mater..

[17] Zhang D., Shao Y. (2016). Effect of early carbonation curing on chloride penetration and weathering carbonation in concrete. Constr. Build. Mater..

[18] (2019). Technical Standard for Application of Lightweight Aggregate Concrete.

[19] (2010). Lightweight Aggregates and Its Test Methods-Part 2: Test Methods for Lightweight Aggregates.

[20] (1986). Test Method for Mechanical Properties of Ordinary Concrete.

[21] (2009). Standard for Test Methods for Long-Term Performance and Durability of Ordinary Concrete.

[22] Smirnov V.G., Manakov A.Y., Dyrdin V.V., Ismagilov Z.R., Mikhailova E.S., Rodionova T.V. (2018). The formation of carbon dioxide hydrate from water sorbed by coals. Fuel.

[23] Wang J., Wang S., Wang H., He Z. (2022). Influence of Ceramsite with Assembly Unit of Sludge and Excavated Soil on the Properties of Cement Concrete. Materials.

[24] Ahmad S., Assaggaf R., Maslehuddin M., Al-Amoudi O., Adekunle S., Ali S.I. (2017). Effects of carbonation pressure and duration on strength evolution of concrete subjected to accelerated carbonation curing. Constr. Build. Mater..

[25] Zhu J., Liu S., Song L., Qu Z., Wang H. (2022). Inflfluence of Carbon Dioxide Curing on the Corrosion Resistance of Reinforced Cement Mortar under the External Erosion of NaCl Freeze–Thaw Cycle. Appl. Sci..

[26] Castellote M., Andrade C., Turrillas X., Campo J., Cuello G.J. (2008). Accelerated carbonation of cement pastes in situ monitored by neutron diffraction. Cem. Concr. Res..

[27] Huang G., Wang H., Shi F. (2021). Coupling Effect of Salt Freeze-thaw Cycles and Carbonation on the Mechanical Performance of Quick Hardening Sulphoalu-minate Cement-based Reactive Powder Concrete with Basalt Fibers. Coatings.

[28] Wang H., Gao X., Liu J. (2018). Effects of salt freeze-thaw cycles and cyclic loading on the piezoresistive properties of carbon nanofibers mortar. Constr. Build. Mater..

[29] Wang H., Gao X., Liu J. (2018). Coupling effect of salt freeze-thaw cycles and cyclic loading on performance degradation of carbon nanofiber mortar. Cold Reg. Sci. Technol..

[30] Liu Q., Iqbal M., Yang J., Lu X., Zhang P., Rauf M. (2021). Prediction of chloride diffusivity in concrete using artificial neural network: Modelling and performance evaluation. Constr. Build. Mater..

[31] Liu X., Chia K., Zhang M. (2011). Water absorption, permeability, and resistance to chloride-ion penetration of lightweight aggregate concrete. Constr. Build. Mater..

[32] Berger R.L., Klemm W. (1972). Accelerated curing of cementitious systems by carbon dioxide: Part II. Hydraulic calcium silicates and aluminates. Cem. Concr. Res..

[33] Merino I., Arevalo L., Romero F. (2007). Preparation and characterization of ceramic products by thermal treatment of sewage sludge ashes mixed with different additives. Waste Manag..

[34] Chen G., Wang K. (2018). Mechanical and thermal properties of glass fibrereinforced ceramsite-foamed concrete. Indoor Built Environ..

[35] Zhang D., Shao Y. (2018). Surface scaling of CO_2_ -cured concrete exposed to freeze-thaw cycles. J. CO_2_ Util..

[36] Ben Mansour H., Dhouibi L., Idrissi H. (2018). Effffect of phosphate-based inhibitor on prestressing tendons corrosion in simulated concrete pore solution contaminated by chloride ions. Constr. Build. Mater..

[37] Wang H., Zhang A., Zhang L., Wang Q., Yang X.-H., Gao X., Shi F. (2020). Electrical and piezoresistive properties of carbon nanofifififiber cement mortar under difffferent temperatures and water contents. Constr. Build. Mater..

[38] Chi L., Wang Z., Lu S., Zhao D., Yao Y. (2019). Development of mathematical models for predicting the compressive strength and hydration process using the EIS impedance of cementitious materials. Constr. Build. Mater..

[39] Zhang Q., Xu W., Sun Y., Ji Y., Cerný R. (2022). Investigation on Mechanical and Microstructure Properties of Steel Fiber Reinforced Concrete. Adv. Mater. Sci. Eng..

[40] Liang J., Zhu H., Chen L., Han X., Guo Q., Gao Y., Liu C. (2019). Rebar corrosion investigation in rubber aggregate concrete via the chloride electro-accelerated test. Materials..

[41] Zhang B., Tan H., Shen W., Xu G., Ma B., Ji X. (2018). Nano-silica and silica fume modifififified cement mortar used as Surface Protection Material to enhance the impermeability. Cem. Concr. Compos..

[42] Berger R., Young J., Leung K. (1972). Acceleration of hydration of calcium silicates by carbon dioxide treatment. Nature.

